# Isolated Optic Neuritis Revealing Lyme Disease in an Adolescent: A Case Report and Literature Review

**DOI:** 10.7759/cureus.98396

**Published:** 2025-12-03

**Authors:** Taoufik Boubga, Amine Hafessi, Brahim Benameur, Naoufal Assoufi

**Affiliations:** 1 Department of Neurology, Military Hospital Oued Eddahab, Centre Hospitalo-Universitaire (CHU) Souss Massa, Agadir, MAR; 2 Department of Internal Medicine, Military Hospital Oued Eddahab, Agadir, MAR; 3 Department of Medicine, Military Hospital Oued Eddahab, Agadir, MAR; 4 Department of Internal Medicine, Military Hospital Oued Eddahab, Medical and Pharmacological Faculty, Mohammed V University, Rabat, MAR

**Keywords:** adolescent, borrelia burgdorferi, case report, ceftriaxone, lyme disease, morocco, neuroborreliosis, optic neuritis

## Abstract

Lyme disease, caused by *Borrelia burgdorferi* sensu lato, can occasionally involve the nervous system and lead to a variety of neurological manifestations. Ocular complications are uncommon, and optic neuritis (ON) represents one of the rarest presentations. We describe the case of a 17-year-old girl from Morocco who developed progressive visual loss and eye pain on the right side. Ophthalmologic evaluation revealed unilateral optic disc swelling and macular edema, and magnetic resonance imaging confirmed right optic nerve inflammation. Cerebrospinal fluid analysis demonstrated lymphocytic pleocytosis and elevated protein, while serological and intrathecal testing identified *B. burgdorferi *infection. The patient was treated with intravenous ceftriaxone for 21 days without corticosteroids and experienced near-complete recovery of vision. This case highlights the importance of considering Lyme disease in the differential diagnosis of ON, even in regions where it is rarely reported. Early recognition and antibiotic therapy can lead to excellent visual outcomes and prevent misdiagnosis as demyelinating disease.

## Introduction

Lyme borreliosis, caused by *Borrelia burgdorferi* sensu lato, remains the most prevalent vector-borne zoonotic infection across the Northern Hemisphere [1]. The disease is transmitted through the bite of infected Ixodes ticks and may involve multiple organ systems, including the skin, joints, heart, and nervous system. Neurological involvement, referred to as Lyme neuroborreliosis (LNB), develops in approximately 10%-15% of untreated infections and manifests as meningitis, cranial neuropathies (most notably facial palsy), or radiculoneuritis [2].

Ocular involvement in Lyme disease is relatively uncommon, but when present, it may affect virtually any structure of the eye, including the conjunctiva, uvea, retina, or optic nerve. Among these, optic neuritis (ON) represents one of the rarest and most diagnostically challenging neuro-ophthalmic manifestations [3]. In most clinical contexts, ON is associated with demyelinating diseases such as multiple sclerosis (MS), neuromyelitis optica spectrum disorder (NMOSD), or myelin oligodendrocyte glycoprotein antibody disease (MOGAD). Infectious or inflammatory causes, including Borrelia infection, may therefore be overlooked, especially in regions where Lyme disease is considered rare.

In this report, we present a case of isolated unilateral ON as the first manifestation of early neuroborreliosis in an adolescent from Morocco, a country where human Lyme infection has been scarcely reported. This case emphasizes the importance of maintaining a broad differential diagnosis for ON, as well as the necessity of raising clinical awareness of emerging vector-borne diseases in North Africa.

## Case presentation

A 17-year-old girl from Agadir, Morocco, presented with a two-week history of progressive visual decline in her right eye, accompanied by ocular pain on movement. Her symptoms were preceded by a mild febrile episode (38°C), headache, and transient arthralgia affecting her wrists and knees. She denied diplopia, photophobia, or rash. Although she lived in an urban area, she regularly visited wooded rural regions and frequently interacted with domestic animals. No tick bite or erythema migrans was recalled.

**Figure 1:**
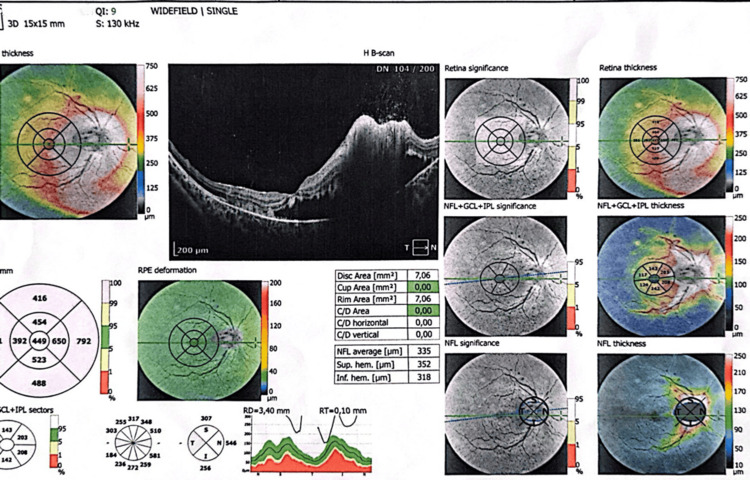
OCT findings OCT of the affected eye showing structural changes consistent with acute optic neuritis and macular swelling OCT: optical coherence tomography

On examination, visual acuity in the right eye was reduced to counting fingers, with a right relative afferent pupillary defect and a dense central scotoma. The left eye maintained normal visual acuity (10/10). Fundoscopic examination showed marked optic disc swelling (grade 3 papilledema) with macular edema, confirmed by optical coherence tomography (OCT). The left eye was normal (Figure 1).

**Figure 2:**
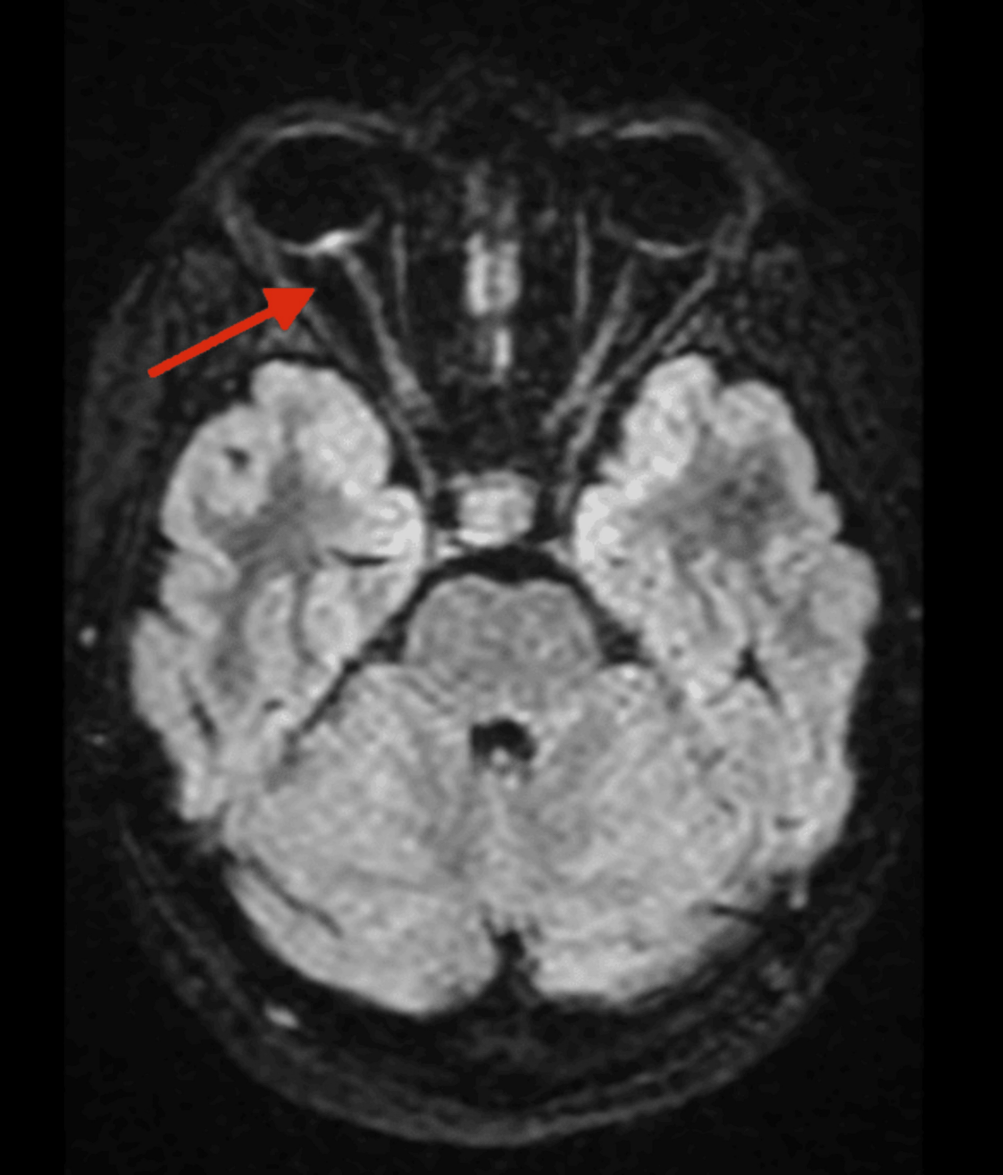
MRI findings Axial and coronal T1-weighted postcontrast fat-suppressed images showing marked enhancement and thickening of the right intraorbital optic nerve, consistent with acute inflammatory neuritis. The left optic nerve, chiasm, and brain parenchyma are normal, with no demyelinating or ischemic lesions

Magnetic resonance imaging (MRI) of the brain and orbits revealed high fluid-attenuated inversion recovery signal intensity at the retinopapillary junction of the right optic nerve, with contrast enhancement and mild protrusion of the nerve head-findings consistent with papillitis (Figure 2). No abnormalities were noted in the contralateral optic nerve, optic chiasm, or brain parenchyma.

Cerebrospinal fluid (CSF) analysis showed lymphocytic pleocytosis (80 cells/mm³), elevated protein (0.85 g/L), and normal glucose levels. Bacterial cultures and the venereal disease research laboratory were negative. Serologic testing via enzyme-linked immunosorbent assay and confirmatory Western blot detected *B. burgdorferi* IgM with negative IgG, consistent with early infection. Intrathecal synthesis of Borrelia-specific antibodies was positive, confirming central nervous system involvement. Tests for aquaporin-4 (AQP4) and MOG antibodies were negative, ruling out NMOSD and MOGAD. Further investigations for tuberculosis, viral infections, and autoimmune diseases were unremarkable (Table 1).

**Table 1:** Laboratory findings of the patient Summary of cerebrospinal fluid and serological results demonstrating lymphocytic inflammation and specific intrathecal antibody synthesis consistent with definite neuroborreliosis CSF: Cerebrospinal fluid; ELISA: enzyme-linked immunosorbent assay; MOG: myelin oligodendrocyte glycoprotein

Parameter	Result	Reference range	Units
CSF white cell count	80 (lymphocytic)	<5	cells/mm³
CSF protein	0.85	0.15-0.45	g/L
CSF glucose	Normal	2.2-4.4	mmol/L
Serum ELISA (Borrelia IgM)	Positive	Negative	-
Serum ELISA (Borrelia IgG)	Negative	Negative	-
Intrathecal Borrelia antibody index	Positive	Negative	-
Aquaporin-4 antibody	Negative	Negative	-
MOG antibody	Negative	Negative	-

The patient was treated with intravenous ceftriaxone (2 g/day) for 21 days, without corticosteroids. Her vision gradually improved, and full recovery was achieved within three weeks. Follow-up visual field testing and clinical examination confirmed resolution of optic disc edema and restoration of normal retinal nerve fiber thickness.

## Discussion

Although Lyme borreliosis is classically described in temperate climates of Europe and North America, recent ecological and climatic shifts have led to its wider geographic spread [1]. In North Africa,* Ixodes ricinus*, the principal European vector, has been detected in the Middle and High Atlas Mountains. Several entomological studies have confirmed the presence of Borrelia DNA in Moroccan tick populations, suggesting that human infection may be underdiagnosed due to limited awareness and diagnostic availability [4].

The optic nerve may be affected in Lyme disease through multiple mechanisms. Direct bacterial invasion of the leptomeninges surrounding the optic nerve can cause vasculitis, demyelination, and subsequent edema [5]. Alternatively, molecular mimicry between Borrelia surface proteins and human myelin antigens, such as myelin basic protein, can induce autoimmune demyelination. Proinflammatory cytokines, including interleukin-6, CXCL13, and tumor necrosis factor-α, have been identified in CSF samples from acute neuroborreliosis cases, implicating immune-mediated damage [6]. These inflammatory processes are usually reversible when promptly treated with antibiotics, explaining the good prognosis of early disease.

According to the European Federation of Neurological Societies (EFNS) guidelines [7], a definite diagnosis of LNB requires neurological symptoms compatible with LNB, CSF pleocytosis, and intrathecal synthesis of Borrelia-specific antibodies. Our patient fulfilled all three criteria. The absence of AQP4 and MOG antibodies and normal brain MRI excluded autoimmune demyelinating etiologies, confirming a diagnosis of definite LNB with isolated optic nerve involvement.

ON as a manifestation of LNB remains an exceptional clinical finding, with fewer than 40 cases described globally [3]. Since its first recognition in the mid-1980s, the condition has been reported sporadically in both adults and children, with variations in laterality, severity, and associated neurological findings. Early documented cases originated in North America and Central Europe, where ON was often part of disseminated neuroborreliosis accompanied by meningitis, radiculitis, or facial palsy [1,3]. Subsequent studies recognized isolated ON as a distinct presentation of LNB, characterized by unilateral or bilateral disc swelling and serologic or CSF evidence of Borrelia infection.

The clinical presentation of Lyme-associated optic neuritis (LAON) is heterogeneous. Most patients present with acute or subacute visual loss, either unilateral or bilateral, often accompanied by eye pain and variable optic disc swelling. Bilateral cases are more likely to occur with meningitic symptoms or cranial neuropathies, whereas unilateral cases, such as our patient’s, tend to represent early or localized infection. Pediatric cases appear to have a higher proportion of isolated ON presentations than adults, possibly due to heightened immune reactivity [8].

MRI findings are variable, with some cases showing enhancement of the intraorbital or intracanalicular optic nerve, while others reveal perineural enhancement. Unlike demyelinating ON associated with MS, typical brain lesions or chiasmatic involvement are usually absent. CSF analysis commonly demonstrates lymphocytic pleocytosis and elevated protein, while intrathecal antibody synthesis remains the diagnostic cornerstone [7].

Pathophysiological studies suggest both direct and immune-mediated mechanisms of neural injury [5,6]. The spirochete’s invasion of the meninges and optic nerve sheath contributes to perineuritis, while immune responses promote secondary demyelination. Cytokine-mediated inflammation, especially CXCL13 elevation, correlates with disease activity and may serve as a biomarker of active neuroborreliosis.

Nearly all published cases of LAON report significant or complete visual recovery following timely antibiotic therapy, most commonly intravenous ceftriaxone for 14-21 days. Some reports describe adjunctive corticosteroid use, but its benefit remains controversial [9]. In most cases, antibiotic monotherapy is sufficient, particularly when treatment is initiated early. For instance, in an adult case of isolated optic neuropathy attributed to *B. burgdorferi*, visual acuity improved dramatically with ceftriaxone alone, supporting the reversibility of infection-driven inflammation [10].

Epidemiologically, most published cases arise from Europe and North America, but emerging reports from Asia, South America, and North Africa suggest global underrecognition. Ecological studies in Morocco and Algeria confirm the presence of *I. ricinus* ticks carrying Borrelia DNA [4], reinforcing the need to consider Lyme disease even in nonendemic regions.

According to the EFNS (2010) and Infectious Diseases Society of America (2020) guidelines [7,11], intravenous ceftriaxone at 2 g daily for 14-21 days remains the treatment of choice for central nervous system involvement, including optic nerve inflammation. Cefotaxime and high-dose penicillin G are acceptable alternatives. Oral doxycycline may suffice for mild peripheral forms but is less effective for central involvement. Corticosteroids, although used empirically, remain controversial due to concerns of delayed bacterial clearance [9]. Our patient’s recovery with antibiotic therapy alone supports the adequacy of monotherapy in early neuroborreliosis.

Prognosis is generally excellent when treatment is initiated promptly, with most patients achieving full visual recovery. Late or bilateral cases may result in partial visual loss or optic atrophy. Recurrence is rare but warrants follow-up to rule out postinfectious demyelination. Long-term monitoring with OCT and MRI ensures stability and exclusion of evolving autoimmune processes [12].

## Conclusions

This case of definite LNB presenting as isolated unilateral ON in an adolescent highlights the need to include *B. burgdorferi* infection in the differential diagnosis of ON, even in nonendemic regions. The combination of clinical findings, CSF abnormalities, and positive intrathecal antibody synthesis permitted an early and accurate diagnosis. Prompt intravenous ceftriaxone therapy without corticosteroids resulted in full recovery. Clinicians should maintain vigilance for Lyme disease in patients presenting with ON and rural exposure, as timely recognition ensures an excellent prognosis and prevents misclassification as a demyelinating disorder.
